# Hepatic Proteomic Changes Associated with Liver Injury Caused by Alcohol Consumption in *Fpr2^−^*^/*−*^ Mice

**DOI:** 10.3390/ijms25189807

**Published:** 2024-09-11

**Authors:** Josiah E. Hardesty, Jeffrey B. Warner, Daniel W. Wilkey, Brett S. Phinney, Michelle R. Salemi, Michael L. Merchant, Craig J. McClain, Dennis R. Warner, Irina A. Kirpich

**Affiliations:** 1 Division of Gastroenterology, Hepatology and Nutrition, Department of Medicine, University of Louisville, Louisville, KY 40202, USA; josiah.hardesty@louisville.edu (J.E.H.); jeff.warner01@gmail.com (J.B.W.); craig.mcclain@louisville.edu (C.J.M.); dennis.warner@louisville.edu (D.R.W.); 2 Department of Pharmacology and Toxicology, School of Medicine, University of Louisville, Louisville, KY 40202, USA; michael.merchant@louisville.edu; 3 The Proteomics Core, School of Medicine, University of Louisville, Louisville, KY 40202, USA; daniel.wilkey@louisville.edu; 4 Proteomics Core Facility, University of California Davis, Davis, CA 95616, USA; bsphinney@ucdavis.edu (B.S.P.); msalemi@ucdavis.edu (M.R.S.); 5 Robley Rex Veterans Medical Center, Louisville, KY 40202, USA; 6 Alcohol Research Center, School of Medicine, University of Louisville, Louisville, KY 40202, USA; 7 Hepatobiology & Toxicology Center, School of Medicine, University of Louisville, Louisville, KY 40202, USA; 8 Department of Microbiology and Immunology, School of Medicine, University of Louisville, Louisville, KY 40202, USA

**Keywords:** alcohol-associated liver disease, FPR2, liver proteome

## Abstract

Alcohol-associated liver disease (ALD) is a prevalent medical problem with limited effective treatment strategies. Although many biological processes contributing to ALD have been elucidated, a complete understanding of the underlying mechanisms is still lacking. The current study employed a proteomic approach to identify hepatic changes resulting from ethanol (EtOH) consumption and the genetic ablation of the formyl peptide receptor 2 (FPR2), a G-protein coupled receptor known to regulate multiple signaling pathways and biological processes, in a mouse model of ALD. Since previous research from our team demonstrated a notable reduction in hepatic FPR2 protein levels in patients with alcohol-associated hepatitis (AH), the proteomic changes in the livers of *Fpr2*^−/−^ EtOH mice were compared to those observed in patients with AH in order to identify common hepatic proteomic alterations. Several pathways linked to exacerbated ALD in *Fpr2*^−/−^ EtOH mice, as well as hepatic protein changes resembling those found in patients suffering from AH, were identified. These alterations included decreased levels of coagulation factors F2 and F9, as well as reduced hepatic levels of glutamate-cysteine ligase catalytic subunit (GCLC) and total glutathione in *Fpr2*^−/−^ EtOH compared to WT EtOH mice. In conclusion, the data suggest that FPR2 may play a regulatory role in hepatic blood coagulation and the antioxidant system, both in a pre-clinical model of ALD and in human AH, however further experiments are required to validate these findings.

## 1. Introduction

Alcohol-associated liver disease (ALD) is the most prevalent disease affecting the liver, and, importantly, there are no existing FDA-approved treatment options [1]. ALD encompasses a spectrum of pathological stages including steatosis, steatohepatitis, fibrosis/cirrhosis, and eventually, hepatocellular carcinoma [2]. The mechanisms governing the pathogenesis of ALD are not well understood, but chronic inflammation combined with impaired resolution and tissue regeneration are phenotypic features of ALD that have moved to the forefront of ALD research [3]. We recently demonstrated that formyl peptide receptor 2 (FPR2) knockout mice fed ethanol (EtOH) developed exacerbated liver injury, inflammation, and compromised regeneration as compared to WT EtOH mice [4]. FPR2 is a G-protein coupled receptor that can be activated by many ligands including Anxa1, fMLF, and resolvin-D1 (RvD1) [5,6]. It has been previously demonstrated that treatment with RvD1 reduced EtOH and lipopolysaccharide (LPS)-induced liver injury and inflammation in mice [7]. Mechanistically, RvD1 attenuated pyroptosis, a type of programmed proinflammatory cell death, in the whole liver, hepatocytes, and macrophages [7]. In addition, RvD1 treatment promoted the macrophage efferocytosis of dead hepatocytes [7], which can further alleviate liver injury and inflammation [8].

Proteomic analysis enables the identification and quantification of numerous proteins within biological samples [9]. Given that proteins are integral to various cellular functions, proteomics serves as a powerful technique for uncovering mechanisms altered by biological or experimental factors [10]. Recently, plasma proteomic analysis has been employed to discover biomarkers and elucidate mechanisms associated with early-stage ALD as well as more advanced EtOH-induced liver injury like alcohol-associated hepatitis (AH) [11,12]. Our group utilized hepatic proteomic analysis to identify mechanisms involved in AH pathogenesis [13] and to explore steroid responsiveness in patients with AH [14]. Our findings revealed significant differences in the liver proteome of patients with AH compared to non-ALD individuals, with a notable downregulation of proteins and biological processes related to hepatic proteostasis and protein synthesis, among others [13]. The aim of the current study was to investigate *Fpr2*-mediated alterations in the hepatic proteome associated with ALD using experimental mice. Additionally, we aimed to identify common hepatic proteomic changes in *Fpr2*^−/−^ EtOH mice and patients with AH.

## 2. Results

### 2.1. Hepatic Proteomic Alterations Associated with Liver Injury in Fpr2^−/−^ Mice Fed EtOH

**Figure 1:**
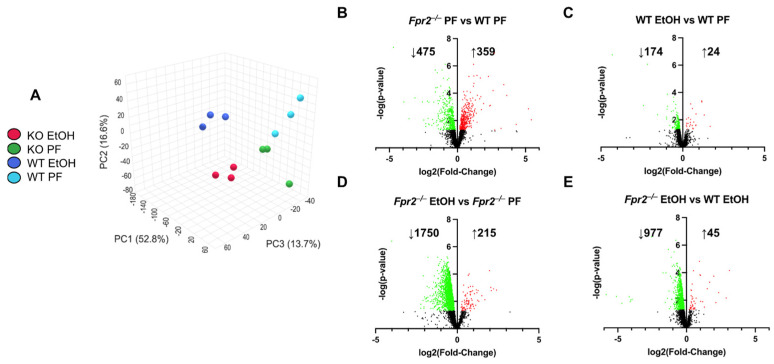
Hepatic proteomic alterations in experimental WT and *Fpr2*^−/−^ mice. (**A**) PCA of the hepatic proteome for WT PF (light blue), WT EtOH (dark blue), *Fpr2*^−/−^ PF (green), and *Fpr2*^−/−^ EtOH (red) experimental groups. (**B**–**E**) Volcano plot analyses for the indicated comparisons and the number of decreased (green) and increased (red) proteins.

The mouse model used for this study was a chronic plus binge EtOH administration, where WT and *Fpr2*^−/−^ mice were placed on either control or EtOH-containing diets ad libitum with a single EtOH bolus at the end of the experiment. As we previously reported, compared to WT, the *Fpr2*^−/−^ mice in this model developed more severe liver injury and inflammation [4], confirmed by elevated plasma ALT, AST, hepatic *Lcn2*, and *Pai1* mRNA expression as compared to WT EtOH mice (Table 1). In addition, *Fpr2*^−/−^ mice had compromised liver regeneration in response to EtOH, in part via mechanisms involved in immune cell dysregulation, such as a reduced number of hepatic monocyte-derived restorative macrophages, likely resulting from impaired differentiation. In the current study, we performed hepatic proteomic analysis to gain deeper insight into the mechanisms contributing to exacerbated liver injury in *Fpr2*^−/−^ mice fed EtOH. This analysis identified 3297 proteins across all of the following experimental groups: WT PF, WT EtOH, *Fpr2*^−/−^ PF, and *Fpr2*^−/−^ EtOH. These groups were well separated when analyzed by PCA, demonstrating a clear effect by the loss of FPR2 expression in the presence or absence of EtOH consumption (Figure 1A). When comparing *Fpr2*^−/−^ PF mice vs. WT PF mice, there were 475 proteins that were decreased and 359 that were increased (Figure 1B). EtOH consumption by WT mice led to an increase in 24 proteins and a decrease in 174 proteins, relative to WT-PF mice (Figure 1C), while in the livers of *Fpr2*^−/−^ mice, those fed EtOH had 1750 proteins that were decreased and 215 that were increased compared to *Fpr2*^−/−^ PF mice (Figure 1D). Finally, the expression of 977 proteins was decreased in *Fpr2*^−/−^ EtOH vs. WT EtOH mice and 45 proteins were increased (Figure 1E). These results demonstrate that *Fpr2*^−/−^ mice developed exacerbated alcohol-induced liver injury and inflammation as compared to WT mice, which was associated with distinct liver proteome changes.

### 2.2. Similarity in Proteomic Changes in the Livers of Patients with AH to Those Found in Fpr2^−/−^ Mice Fed EtOH

**Figure 2:**
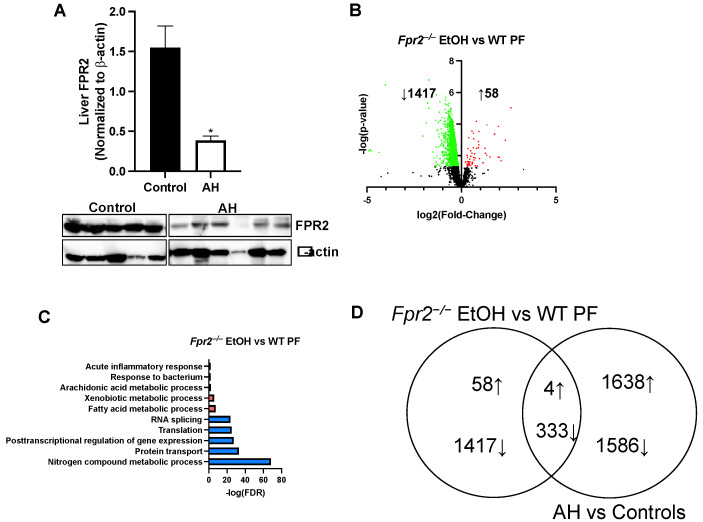
Hepatic proteome changes in human AH that are similar to those in *Fpr2*^−/−^ mice fed EtOH vs. WT PF mice. (**A**) Western blot analysis of FPR2 in human AH and non-ALD control liver samples. Data are presented as the mean ± SEM. * *p* < 0.05 was considered significant. (**B**) Volcano plot analysis for *Fpr2*^−/−^ EtOH vs. WT PF hepatic proteomic changes (green—decreased, red—increased). (**C**) Biological processes representative of proteomic changes in *Fpr2*^−/−^ EtOH vs. WT PF (pink—increased, blue—decreased). (**D**) Venn diagram showing the number of shared hepatic proteomic changes between *Fpr2*^−/−^ EtOH and WT PF and human AH and non-ALD controls.

Previously, we identified significant hepatic proteomic changes in patients with AH vs. non-ALD controls [13]. Here, we have confirmed the decrease in hepatic FPR2 expression in human AH by western blot analysis (Figure 2A). In the current animal study, we used *Fpr2*^−/−^ mice since we found that FPR2 was not reduced by EtOH in WT mice. This lack of a reduction is likely due to the relatively mild pathological features of ALD observed in our animal model, in contrast to the more severe liver injury seen in human AH, where FPR2 levels are significantly decreased (Figure 2A). Therefore, hepatic proteomic changes that are common between *Fpr2*^−/−^ mice fed EtOH and human AH may shed unique mechanistic insights into AH pathogenesis in humans. An analysis of hepatic proteome in mice revealed that there were 1417 downregulated and 58 upregulated proteins when comparing *Fpr2*^−/−^ EtOH vs. WT PF mice, as visualized by volcano plot analysis (Figure 2B). GO biological process analysis identified five upregulated processes in this comparison, which included fatty acid, xenobiotic and arachidonic acid metabolism, and the response to bacterium and acute inflammatory response (Figure 2C). The GO biological processes for the other comparisons can be found in (Figure S1). There were also five downregulated GO biological processes in *Fpr2*^−/−^ EtOH vs. WT PF mice, which included nitrogen metabolism, protein transport, the regulation of gene expression, translation, and RNA splicing (Figure 2C). Further, when comparing these liver proteomic changes in mice to hepatic proteomic changes in human AH, there were 337 commonly shared proteins (4 upregulated and 333 downregulated, Figure 2D). These data suggest that there are several hepatic proteomic changes in *Fpr2*^−/−^ EtOH vs. WT PF mice that are similar to human AH vs. non-ALD.

**Figure 3:**
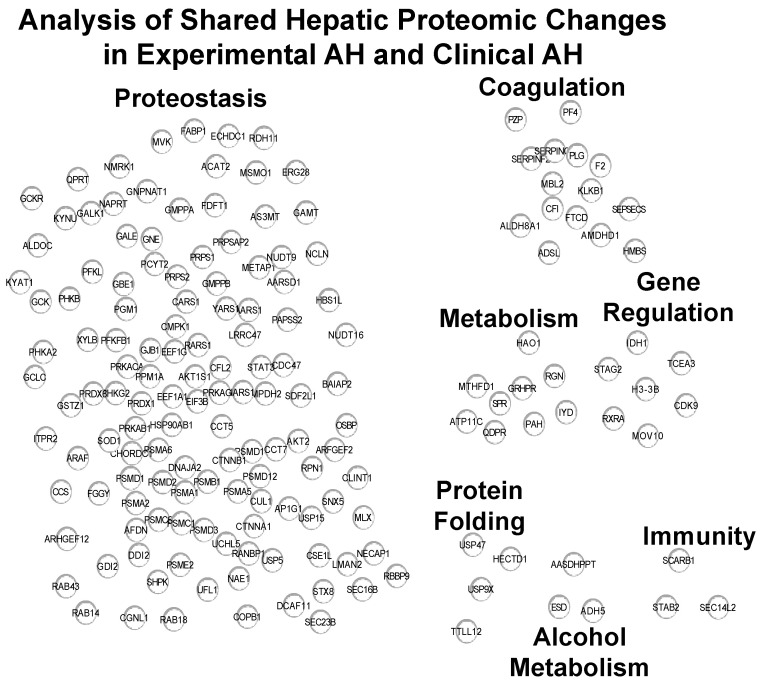
Analysis of shared hepatic proteomic changes in experimental and clinical AH. Protein cluster analysis of the shared proteins between *Fpr2*^−/−^ EtOH and WT PF and human AH and non-ALD controls that were decreased.

The four hepatic proteins that were upregulated in human AH and *Fpr2*^−/−^ EtOH mice, relative to non-ALD and WT-PF controls, respectively, included lipocalin-2 (LCN2), antioxidant 1 copper chaperone (ATOX1), cathepsin L1 (CTSL), and NADPH quinone dehydrogenase 1 (NQO1). Protein cluster analysis on the 333 shared proteins that were downregulated revealed that the downregulated processes included proteostasis, coagulation, metabolism, gene regulation, protein folding, alcohol metabolism, and immunity (Figure 3).

Taken together, these data suggest that the biological processes disrupted in EtOH-fed *Fpr2*^−/−^ mice are similar to those observed in human AH, highlighting their potential relevance to this pathology.

### 2.3. Analysis of Selected Proteomic Responses Relevant to Human Disease, Which Were Common in Experimental ALD and Clinical AH

#### 2.3.1. Hepatic Blood Coagulation Gene and Protein Expression Are Reduced in *Fpr2^−/−^* EtOH vs. WT EtOH Mice

**Figure 4:**
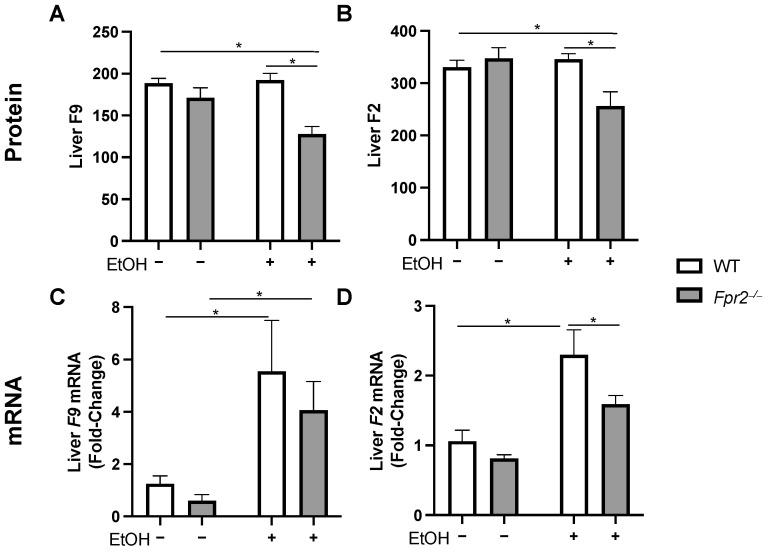
Hepatic blood coagulation gene and protein expression are reduced in *Fpr2*^−/−^ EtOH vs. WT EtOH mice. (**A**,**B**) Protein and (**C**,**D**) mRNA expression of F9 and F2, respectively. Data are presented as the mean ± SEM. * *p* < 0.05 was considered significant.

Among the downregulated proteins were F2 and F9, which are functional mediators of blood coagulation, a process often compromised in ALD/AH [15]. We further investigated these factors due to their relevance to human ALD/AH pathogenesis. Thus, hepatic F9 (Figure 4A) and F2 (Figure 4B) protein expression was significantly reduced in *Fpr2*^−/−^ EtOH vs. WT EtOH mice, but there was no EtOH effect when comparing WT EtOH vs. WT PF or *Fpr2*^−/−^ EtOH vs. *Fpr2*^−/−^ PF. In contrary, both *F9* (Figure 4C) and *F2* (Figure 4D) were elevated by EtOH at the gene levels in both genotypes, but to a lesser extent in *Fpr2*^−/−^ mice. These data suggest that the loss of *Fpr2* may lead to a reduced expression of hepatic F9 and F2 at the gene and protein level, likely exacerbating coagulopathy in ALD. Therefore, FPR2 might serve as a potential mediator of blood coagulation.

#### 2.3.2. *Fpr2^−/−^* EtOH Mice Have Reduced Hepatic GSH as Compared to WT EtOH Mice

**Figure 5:**
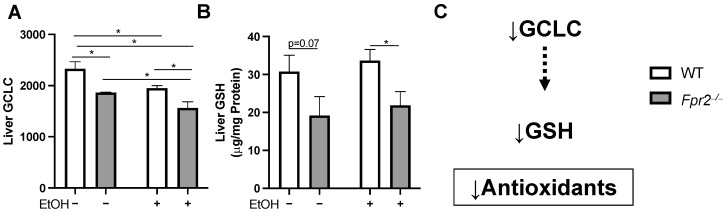
*Fpr2*^−/−^ EtOH mice have reduced hepatic GSH as compared to WT EtOH mice. (**A**) Liver GCLC protein expression in experimental groups. (**B**) Total liver GSH levels among experimental groups. (**C**) Model illustrating GCLC-mediated synthesis of the hepatic antioxidant glutathione (GSH). Data are presented as the mean ± SEM. * *p* < 0.05 was considered significant.

Another downregulated protein of interest was the glutamate-cysteine ligase catalytic subunit (GCLC, Figure 5A), an enzyme involved in the synthesis of the antioxidant, glutathione (GSH) [16]. Since we found a reduced expression of GCLC, the rate-limiting enzyme subunit for GSH synthesis, total GSH levels in the liver were measured. Total liver GSH levels were reduced in *Fpr2*^−/−^ mice (PF or EtOH-fed) and were significantly reduced in *Fpr2*^−/−^ EtOH vs. WT EtOH mice (Figure 5B). Collectively, these data indicate that FPR2 may help maintain the liver antioxidant capacity via the regulation of hepatic GSH synthesis (Figure 5C) and the alcohol-mediated dysregulation of GSH levels may contribute to oxidative stress in ALD.

## 3. Discussion

In this study, we identified hepatic proteomic changes that were associated with the loss of FPR2 expression in a pre-clinical animal model of ALD. Additionally, we found that some of these proteomic alterations resembled those found in patients with AH, which also exhibit a reduction in FPR2 expression [13], underscoring the clinical significance of the FPR2-mediated proteomic changes in ALD.

Among the proteins elevated in both patients with AH and *Fpr2*^−/−^ EtOH mice was LCN2, a pro-inflammatory acute phase protein [17]. It has been previously reported that *Lcn2* knockout mice are protected from alcohol-induced steatosis and liver injury [18]. NQO1 was another hepatic protein whose expression was elevated in both human AH and *Fpr2*^−/−^ EtOH mice. NQO1 levels are elevated in the event of oxidative stress [19], which is a common feature of ALD leading to hepatic damage [20]. Therefore, the loss of FPR2 may enhance oxidative stress via the upregulation of NQO1. Elevated CTSL, a pro-inflammatory protease [21] associated with liver fibrosis [22], was also found in both human AH and *Fpr2*^−/−^ EtOH mice. Previously, acetalaldehyde exposure was shown to reduce CTSL activity [23] in hepatocytes, suggesting the CTSL upregulation observed in this study may compensate for the deleterious impact of alcohol metabolites such as acetaldehyde on CTSL activity. In addition, ATOX1, a copper chaperone protein [24], was also elevated in both human AH and *Fpr2*^−/−^ EtOH mice. Elevated hepatic ATOX1 expression can be indicative of increased copper levels in the liver [25], which is toxic and has been shown to occur in ALD patients [26]. Copper can reduce FPR2-mediated chemotaxis and ligand binding in vitro [27], suggesting that reduced FPR2 in AH may be further exacerbated by ATOX1 upregulation and copper level elevation. Another important clinical feature of AH is impaired blood clotting due to the impaired production of coagulation factors that are synthesized by the liver (e.g., factor IX and prothrombin) [28]. The levels of two specific proteins of the coagulation cascade, F2 and F9, were found to be decreased both in human AH and *Fpr2*^−/−^ EtOH mice, potentially further exacerbating the defects in this cascade. FPR2 has been previously implicated in blood coagulation via platelet and immune cell aggregation [29], and our findings suggest that it may also play a more direct role via positively regulating the expression of coagulation factors synthesized by the liver. Lastly, the liver antioxidant capacity is highly dependent on normal levels of GSH [30], which are depleted in ALD [30]. Interestingly, treatment with RvD1, an FPR2 ligand, enhanced GSH levels in WT but not *Fpr2*^−/−^ macrophages [31]. Our data suggest that liver GSH levels may be decreased due to a reduction in GCLC, the rate-limiting enzyme subunit for GSH synthesis [32]. FPR2 was also shown to elevate the expression of *Nrf2*, a transcriptional regulator of the antioxidant response element in an animal model of steatohepatitis [33]. Therefore, FPR2 potentially regulates GSH synthesis in multiple cell types via the transcriptional regulation and expression of GCLC.

The current study has several strengths and limitations. A notable strength was the use of an unbiased proteomic approach, which facilitated the discovery of novel mechanisms of ALD/AH potentially regulated by FPR2. Additionally, this study employed a translational approach, highlighting several consistent proteomic changes between clinical and pre-clinical ALD settings. However, weaknesses include the associative nature of the identified mechanisms, which require further investigation to establish definitive causal relationships. Another limitation was the study’s focus primarily on changes resulting from the absence of FPR2, without an exploration of downstream signaling mechanisms regulated by distinct FPR2 ligands [34]. Additionally, the proteomic analysis was limited to a relatively small sample size (n = 3), although the PCA plot demonstrated consistent protein changes across the samples (Figure 1A). Lastly, since the mice used for this study were whole-body knockout, we cannot rule out extra hepatic effects of *Fpr2* gene knockout that may influence the observed results.

In summary, our findings indicate that there are some similarities in hepatic proteome changes between EtOH-induced liver injury in *Fpr2*-deficient mice and AH in humans, where FPR2 expression is also diminished. This suggests a potential significant role for FPR2 in the pathology of ALD in humans. However, it remains unclear whether the loss of FPR2 expression in humans is a causative factor or merely associated with the development of AH, warranting further investigation.

## 4. Materials and Methods

### 4.1. Animal Model

WT and *Fpr2*^−/−^ mice were treated as described previously in a chronic plus binge model of experimental ALD [4]. Briefly, 8- to 10-week-old female *Fpr2*^−/−^ and WT littermate mice were fed Lieber-DeCarli EtOH-containing or isocaloric control diets for 4 weeks, followed by either a water or EtOH binge (5 g/kg) on the final day of the experiment. The mice were then euthanized 9 h after the binge, and liver tissues were collected and stored at −80 °C for the future analyses.

### 4.2. Proteomic Sample Preparation

Mouse liver tissue (~100 mg) was homogenized in modified RIPA buffer (50 mM Tris, 150 mM NaCl, 0.1% Triton X-100, 0.5% sodium deoxycholate, 0.1% SDS) supplemented with protease and phosphatase inhibitors (Halt™, Thermo Fisher Scientific, Waltham, MA, USA) in tubes filled with 0.5 mm glass beads (BioSpec, Bartlesville, OK, USA) using a BioSpec Mini BeadBeater. After centrifugation at 16,000× *g*, supernatants were collected and protein concentrations were assessed by a bicinchoninic acid (BCA) assay. Liver protein extracts (250 µg) were digested according to the S-trap protocol (Protifi, LLC, Fairport, NY, USA), as previously reported [35], with peptide recovery estimated by a pierce quantitative colorimetric peptide assay (ThermoFisher Scientific, Waltham, MA, USA). Digested peptides (100 µg) were labeled with TMTpro 16Plex (ThermoFisher Scientific, Waltham, MA, USA) reagents, admixed into a single pool, and then desalted using an Oasis HLB 1cc (30 mg) Cartridge (Waters Corporation, Milford, MA, USA). Prior to liquid chromatography–mass spectrometric (LC-MS) analysis, the samples were fractionated using a U3000SD uHPLC system (ThermoFisher Scientific, Waltham, MA, USA) and a Waters BEH XBridge C18 5 µm 3.0 × 150 mm column. Fractions were collected in 1.25 min intervals over a gradient of solvents A = 5% *v*/*v* acetonitrile/10 mM ammonium formate pH 10 and B = 90% *v*/*v* acetonitrile/10 mM ammonium formate pH 10 (0% to 40% B over 65 min at a flow rate of 300 µL/min). A total of 54 collected fractions were concatenated into 18 by pooling three fractions (fractions n, n + 18, n + 36) plus the final fraction collected from the end of the LC separation.

### 4.3. LC-MS Data Collection

The fractions were dried in a SpeedVac (ThermoFisher Scientific, Waltham, MA, USA) and submitted for LC-MS analysis at the University of California Davis Genome Center Proteomics Core Facility. Briefly, the samples were separated on an RSLCNano system at 500 nL/min with a 70 min gradient from 3% to 55% acetonitrile/0.05% formic acid gradient. Eluates were introduced into an Orbitrap Fusion Lumos mass spectrometer (ThermoFisher Scientific, Waltham, MA, USA) system with the ion transfer capillary temperature and spray voltage of the mass spectrometer set at 275 °C and 1.8 kV, respectively. The MS was operating in positive polarity and collected data using a TMT SPS MS3 method with a 3 s cycle time. An Orbitrap MS1 master scan (normal mass range; 120,000 resolution, AGC target 4E5, profile data type) was obtained using the range 400–1400 *m*/*z*. MS2 scans (rapid scan rate, AGC target 2E4, centroid data type) were obtained in the ion trap on peaks that had a minimum signal threshold of 5000 counts. Multi-notch (10 notches) Orbitrap MS3 scans (50,000 resolution, AGC target 1E5, centroid data type) were obtained using the range 100–500 *m*/*z*.

### 4.4. Proteomic Data Analysis

The collected data analysis was directed through Proteome Discoverer v2.4.0.305 (ThermoFisher Scientific, Waltham, MA, USA) using SequestHT and the 1/29/2021 version of the UniprotKB reviewed canonical Mus musculus sequences (Proteome ID UP000000589), considering trypsin (KR|P) digestion with up to two missed cleavages, the dynamic modifications Oxidation (M), Acetyl (Protein N-term), Met-los (Protein N-term), and Met-loss+Acetyl (Protein N-term), and the static modifications Carbamidomethyl (C) and TMT pro (K, any N-term). The precursor and fragment mass tolerances were 10 ppm and 0.6 Da, respectively. In the consensus step, proteins were quantified from the summed abundances of all high-confidence unique and razor reporter ion abundances. The samples were normalized to the total peptide amount and scaled to 100 on average. The proteins were grouped by the strict parsimony principle. Peptides and proteins were accepted at 1% FDR for high confidence or 5% for medium confidence based on the *q*-value. A protein text file was exported from the consensus workflow result of Proteome Discoverer for curation in Microsoft Excel version 16.88. In total, 3307 unique proteins were identified in WT PF and *Fpr2*^−/−^ PF mice, 3308 were identified in WT EtOH mice, and 3302 were identified in *Fpr2*^−/−^ EtOH mice.

### 4.5. Cytoscape and Principal Component Analysis

Differentially expressed proteins were analyzed in Cytoscape to identify protein clusters and the associated Gene Ontology Biological Process [13]. Principal component analysis (PCA) was performed in Metaboanalyst (https://www.metaboanalyst.ca/, version 5.0, last accessed on 5 May 2023) [36].

### 4.6. Western Blot Analysis

Human liver tissue samples were homogenized in RIPA buffer (10 mM Tris-HCl, pH 8.0, 1 mM EDTA, 0.5 mM EGTA, 1% Triton X-100, 0.1% sodium deoxycholate, 0.1% SDS, and 140 mM NaCl) supplemented with protease and phosphatase inhibitors (HALT^™^, Thermo Fisher Scientific), followed by centrifugation for 10 min at 16,000× *g*. Protein concentrations were determined by the bicinchoninic acid assay (BCA) (Pierce^™^ BCA protein assay kit, Thermo Fisher Scientific). The proteins were separated on Criterion TGX Any kDa gels (BioRad, Hercules, CA, USA) and then transferred to PVDF membranes and blocked in 5% milk TBS + 0.1% Tween-20. The membranes were incubated overnight at 4 °C with primary antibodies, thoroughly washed, and then incubated with secondary antibodies at room temperature for 1 h and washed, and signals were developed with ECL substrate (Clarity Max, BioRad) and imaged via the ChemiDoc instrument (BioRad). Band densitometric analysis was conducted with ImageLab software, version 6.0.1 (BioRad). The primary antibodies FPR2 were purchased from Thermo-Scientific (Waltham, MA, USA), and β-actin was purchased from Cell Signaling Technology (Danvers, MA, USA). Secondary antibodies were purchased from ThermoFisher Scientific.

### 4.7. RT qPCR Analysis

Liver RNA was extracted using Trizol (ThermoFisher Scientific), followed by cDNA synthesis via the Quantabio qScript cDNA supermix reagent (QuantaBio, Beverly, MA, USA). RT-qPCR was performed on the BioRad CFX384 (Hercules, CA, USA) instrument, and relative gene expression values were calculated via the 2^−ΔΔCt^ method using 18S as the reference gene. The primer sequences can be found in Supplementary Table S1.

### 4.8. Liver Total GSH Measurement

Total GSH was measured in liver extracts via the following commercially available kit from Sigma-Aldrich (St. Louis, MO, USA).

### 4.9. Plasma ALT and AST Measurement

Plasma ALT and AST activity were determined using Infinity (ThermoFisher Scientific) and Pointe Scientific (Fisher Scientific) liquid reagents, respectively, as per the manufacturer’s instructions.

### 4.10. Statistical Analysis

All quantitative data were compared by a one-way ANOVA with a Tukey multiple group comparison test. Statistical significance was denoted by a *p* < 0.05.

## 5. Conclusions

In conclusion, we identified hepatic proteomic markers associated with ALD in *Fpr2*^−/−^ EtOH mice. Several of these hepatic proteomic changes are also found in human AH, suggesting their clinical relevance. The findings from this study highlight the beneficial role of FPR2 in positively regulating the expression of blood coagulation factors and the synthesis of GSH during ALD.

## Figures and Tables

**Table 1:** Alcohol-induced liver injury and inflammation in *Fpr2*^−/−^ mice fed EtOH.

	WT PF	*Fpr2*^−/−^ PF	WT EtOH	*Fpr2*^−/−^ EtOH
Plasma ALT	38.95 ± 3.44	32.38 ± 2.66 ^a^	58.18 ± 5.04 ^a^	144.4 ± 44.42
Plasma AST	206.10 ± 13.85	206.90 ± 16.25	259.30 ± 11.60	432.10 ± 137.70
Liver *Lcn2* mRNA	2.22 ± 0.90 ^a^	5.79 ± 2.85 ^a^	149.40 ± 69.03	354.50 ± 138.80
Liver *Pai1* mRNA	0.98 ± 0.25 ^a^	0.68 ± 0.06 ^a^	1.00 ± 0.25 ^a^	7.53 ± 2.70

Significance (*p* < 0.05) is denoted as follows: a- vs. *Fpr2*^−**/**−^ EtOH. EtOH, ethanol; PF, pair fed; *Lcn2*, lipocalin 2; *Pai1*, plasminogen activator inhibitor-1.

## Data Availability

Proteomics data files for a project entitled “Hepatic proteomic changes associated with liver injury caused by alcohol consumption in *Fpr2*^−/−^ mice” were deposited in the MassIVE (http://massive.ucsd.edu/) accessed on 6 September 2024) data repository (MassIVE identifier MSV000095760) maintained by the Center for Computational Mass Spectrometry at the University of California, San Diego. These data include (A) the primary data files (.RAW) for the 2DLC-MS fractionated mouse liver TMTPro-labeled tryptic lysates, (B) an excel file with assembled Proteome Discoverer v2.4 search results, (C) the sample key, and (D) the UniprotKB reviewed canonical Mus musculus (Proteome ID UP000000589) sequence database. The shared data will be released from private embargo for public access upon acceptance for publication.

## Supplementary Materials

The following supporting information can be downloaded at: https://www.mdpi.com/article/10.3390/ijms25189807/s1.

## References

[1] Seitz H.K., Bataller R., Cortez-Pinto H., Gao B., Gual A., Lackner C., Mathurin P., Mueller S., Szabo G., Tsukamoto H. (2018). Alcoholic liver disease. Nat. Rev. Dis. Primers.

[2] Celli R., Zhang X. (2014). Pathology of Alcoholic Liver Disease. J. Clin. Transl. Hepatol..

[3] Sun J., Zhao P., Shi Y., Li Y. (2023). Recent insight into the role of macrophage in alcohol-associated liver disease: A mini-review. Front. Cell Dev. Biol..

[4] Hardesty J.E., Warner J.B., Song Y.L., Floyd A., McClain C.J., Warner D.R., Kirpich I.A. (2023). Fpr2(-/-) Mice Developed Exacerbated Alcohol-Associated Liver Disease. Biology.

[5] He H.Q., Ye R.D. (2017). The Formyl Peptide Receptors: Diversity of Ligands and Mechanism for Recognition. Molecules.

[6] Chen T., Xiong M., Zong X., Ge Y., Zhang H., Wang M., Won Han G., Yi C., Ma L., Ye R.D. (2020). Structural basis of ligand binding modes at the human formyl peptide receptor 2. Nat. Commun..

[7] Hardesty J.E., Warner J.B., Song Y.L., Rouchka E.C., McClain C.J., Warner D.R., Kirpich I.A. (2023). Resolvin D1 attenuated liver injury caused by chronic ethanol and acute LPS challenge in mice. FASEB J..

[8] Shi H., Moore M.P., Wang X., Tabas I. (2024). Efferocytosis in liver disease. JHEP Rep..

[9] Cho W.C. (2007). Proteomics technologies and challenges. Genom. Proteom. Bioinform..

[10] Al-Amrani S., Al-Jabri Z., Al-Zaabi A., Alshekaili J., Al-Khabori M. (2021). Proteomics: Concepts and applications in human medicine. World J. Biol. Chem..

[11] Argemi J., Kedia K., Gritsenko M.A., Clemente-Sanchez A., Asghar A., Herranz J.M., Liu Z.X., Atkinson S.R., Smith R.D., Norden-Krichmar T.M. (2022). Integrated Transcriptomic and Proteomic Analysis Identifies Plasma Biomarkers of Hepatocellular Failure in Alcohol-Associated Hepatitis. Am. J. Pathol..

[12] Niu L., Thiele M., Geyer P.E., Rasmussen D.N., Webel H.E., Santos A., Gupta R., Meier F., Strauss M., Kjaergaard M. (2022). Noninvasive proteomic biomarkers for alcohol-related liver disease. Nat. Med..

[13] Hardesty J., Day L., Warner J., Warner D., Gritsenko M., Asghar A., Stolz A., Morgan T., McClain C., Jacobs J. (2022). Hepatic Protein and Phosphoprotein Signatures of Alcohol-Associated Cirrhosis and Hepatitis. Am. J. Pathol..

[14] Hardesty J., Hawthorne M., Day L., Warner J., Warner D., Gritsenko M., Asghar A., Stolz A., Morgan T., McClain C. (2024). Steroid responsiveness in alcohol-associated hepatitis is linked to glucocorticoid metabolism, mitochondrial repair, and heat shock proteins. Hepatol. Commun..

[15] Ragni M.V., Lewis J.H., Spero J.A., Hasiba U. (1982). Bleeding and coagulation abnormalities in alcoholic cirrhotic liver disease. Alcohol. Clin. Exp. Res..

[16] Lu S.C. (2009). Regulation of glutathione synthesis. Mol. Aspects Med..

[17] Jaberi S.A., Cohen A., D’Souza C., Abdulrazzaq Y.M., Ojha S., Bastaki S., Adeghate E.A. (2021). Lipocalin-2: Structure, function, distribution and role in metabolic disorders. Biomed. Pharmacother..

[18] Cai Y., Jogasuria A., Yin H., Xu M.J., Hu X., Wang J., Kim C., Wu J., Lee K., Gao B. (2016). The Detrimental Role Played by Lipocalin-2 in Alcoholic Fatty Liver in Mice. Am. J. Pathol..

[19] Ross D., Siegel D. (2021). The diverse functionality of NQO1 and its roles in redox control. Redox Biol..

[20] Tan H.K., Yates E., Lilly K., Dhanda A.D. (2020). Oxidative stress in alcohol-related liver disease. World J. Hepatol..

[21] Ruiz-Blázquez P., Pistorio V., Fernández-Fernández M., Moles A. (2021). The multifaceted role of cathepsins in liver disease. J. Hepatol..

[22] Manchanda M., Das P., Gahlot G.P.S., Singh R., Roeb E., Roderfeld M., Datta Gupta S., Saraya A., Pandey R.M., Chauhan S.S. (2017). Cathepsin L and B as Potential Markers for Liver Fibrosis: Insights From Patients and Experimental Models. Clin. Transl. Gastroenterol..

[23] New-Aaron M., Thomes P.G., Ganesan M., Dagur R.S., Donohue T.M., Kusum K.K., Poluektova L.Y., Osna N.A. (2021). Alcohol-Induced Lysosomal Damage and Suppression of Lysosome Biogenesis Contribute to Hepatotoxicity in HIV-Exposed Liver Cells. Biomolecules.

[24] Hatori Y., Lutsenko S. (2016). The Role of Copper Chaperone Atox1 in Coupling Redox Homeostasis to Intracellular Copper Distribution. Antioxidants.

[25] Gerosa C., Fanni D., Congiu T., Piras M., Cau F., Moi M., Faa G. (2019). Liver pathology in Wilson’s disease: From copper overload to cirrhosis. J. Inorg. Biochem..

[26] Rodríguez-Moreno F., González-Reimers E., Santolaria-Fernández F., Galindo-Martín L., Hernandez-Torres O., Batista-López N., Molina-Perez M. (1997). Zinc, copper, manganese, and iron in chronic alcoholic liver disease. Alcohol..

[27] Kim S.Y., Zhang F., Gong W., Chen K., Xia K., Liu F., Gross R., Wang J.M., Linhardt R.J., Cotten M.L. (2018). Copper regulates the interactions of antimicrobial piscidin peptides from fish mast cells with formyl peptide receptors and heparin. J. Biol. Chem..

[28] Kujovich J.L. (2015). Coagulopathy in liver disease: A balancing act. Hematol. Am. Soc. Hematol. Educ. Program..

[29] Vital S.A., Becker F., Holloway P.M., Russell J., Perretti M., Granger D.N., Gavins F.N. (2016). Formyl-Peptide Receptor 2/3/Lipoxin A4 Receptor Regulates Neutrophil-Platelet Aggregation and Attenuates Cerebral Inflammation: Impact for Therapy in Cardiovascular Disease. Circulation.

[30] Vairetti M., Di Pasqua L.G., Cagna M., Richelmi P., Ferrigno A., Berardo C. (2021). Changes in Glutathione Content in Liver Diseases: An Update. Antioxidants.

[31] Filiberto A.C., Ladd Z., Leroy V., Su G., Elder C.T., Pruitt E.Y., Hensley S.E., Lu G., Hartman J.B., Zarrinpar A. (2022). Resolution of inflammation via RvD1/FPR2 signaling mitigates Nox2 activation and ferroptosis of macrophages in experimental abdominal aortic aneurysms. FASEB J..

[32] Chen Y., Shertzer H.G., Schneider S.N., Nebert D.W., Dalton T.P. (2005). Glutamate cysteine ligase catalysis: Dependence on ATP and modifier subunit for regulation of tissue glutathione levels. J. Biol. Chem..

[33] Li J., Deng X., Bai T., Wang S., Jiang Q., Xu K. (2020). Resolvin D1 mitigates non-alcoholic steatohepatitis by suppressing the TLR4-MyD88-mediated NF-κB and MAPK pathways and activating the Nrf2 pathway in mice. Int. Immunopharmacol..

[34] Lee C., Han J., Jung Y. (2023). Formyl peptide receptor 2 is an emerging modulator of inflammation in the liver. Exp. Mol. Med..

[35] Turaga S.M., Sardiu M.E., Vishwakarma V., Mitra A., Bantis L.E., Madan R., Merchant M.L., Klein J.B., Samuel G., Godwin A.K. (2023). Identification of small extracellular vesicle protein biomarkers for pediatric Ewing Sarcoma. Front. Mol. Biosci..

[36] Xia J., Psychogios N., Young N., Wishart D.S. (2009). MetaboAnalyst: A web server for metabolomic data analysis and interpretation. Nucleic Acids Res..

