# Supplementary material for: Hepatic Proteomic Changes Associated with Liver Injury Caused by Alcohol Consumption in Fpr2−/− Mice
Source: Int J Mol Sci. 2024 Sep 11;25(18):9807. doi: 10.3390/ijms25189807 (PMC11432144; doi:10.3390/ijms25189807)

Supplemental Material

Hepatic proteomic changes associated with liver injury caused by alcohol consumption in *Fpr2*$^{-/-}$ mice

Josiah E. Hardesty[1,2], Jeffrey B. Warner[1,2], Daniel W. Wilkey[3], Brett Phinney[4], Michelle Salemi[4], Michael L. Merchant[2,3], Craig J. McClain[1,2,5,6,7], Dennis R. Warner[1], Irina A. Kirpich [6,7,8*]

Table S1: qPCR Primers

Gene	Forward Primer Sequence	Reverse Primer Sequence
Lcn2	GGAGCGATCAGTTCCGGG	CTGATCCAGTAGCGACAGCC
Pai1	TCAATGACTGGGTGGAAAGG	AGGCGTGTCAGCTCGTCTAC
F2	ATGAGATACAGCCCAGCGTC	CGGTTGTTAAAGGGGCTCTTC
F9	ATGCTGGTGCCAAGTTGGAT	TCCCACATGGAAATGGAACTGT
18S	CTCAACACGGGAAACCTCAC	CGCTCCACCAACTAAGAACG

Figure S1: GO Biological Processes altered in experimental mice

The top biological processes enriched in *Fpr2*$^{-/-}$ PF vs WT PF mice included carbohydrate, lipid, alcohol, purine, and steroid metabolism while the decreased included peptide metabolism, translation, fatty acid metabolism, mt translation, and xenobiotic metabolism (**Fig. S1A**). In the WT EtOH vs WT PF comparison, the enriched processes included lipid, xenobiotic, carboxylic acid metabolism, and the epoxygenase pathway, and lipid biosynthesis while the decreased processes were RNA processing, mRNA splicing, ribonucleoprotein assembly, unsaturated fatty acid metabolism, and hormone metabolism (**Fig. S1B**). The processes increased in *Fpr2*$^{-/-}$ EtOH vs WT PF mice included fatty acid, xenobiotic, arachidonic acid metabolism, and response to bacterium, and acute inflammatory response while the top decreased were nitrogen metabolism, protein transport, PTM regulation of gene expression, translation, and RNA splicing (**Fig. S1C**). In *Fpr2*$^{-/-}$ EtOH vs WT EtOH mice the enriched processes were carboxylic acid, fatty acid, xenobiotic, stress response to metal ion, and the epoxygenase pathway while the decreased processes included nitrogen metabolism, vesicle transport, peptide transport, proteolysis, and blood coagulation (**Fig. S1D**).

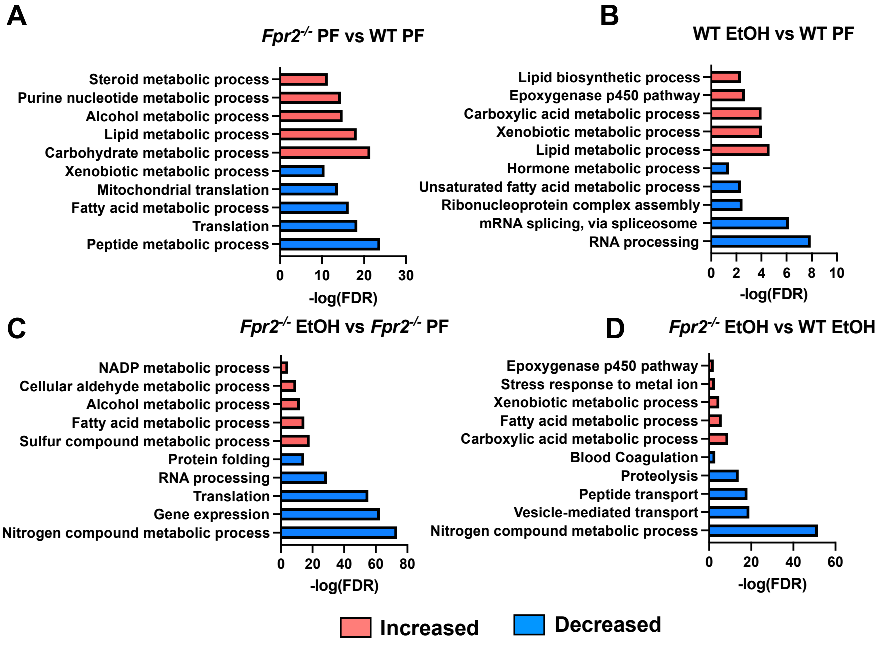

Supplement: Supplementary file 1 [file ijms-25-09807-s001.zip › ijms-3190777-supplementary.pdf]
